# Short-term memory, attention, and temporal orientation as predictors of the cognitive impairment in older adults: A cross-sectional observational study

**DOI:** 10.1371/journal.pone.0261313

**Published:** 2021-12-20

**Authors:** Isabel Gómez-Soria, Chelo Ferreira, Bárbara Oliván Blazquez, Rosa Mª Magallón Botaya, Estela Calatayud

**Affiliations:** 1 Faculty of Health Sciences, Department of Physiatry and Nursing, University of Zaragoza, Zaragoza, Spain; 2 Institute of Health Research of Aragón (IIS Aragón), Zaragoza, Spain; 3 Faculty of Veterinary Sciences, Department of Applied Mathematics and IUMA, University of Zaragoza, Zaragoza, Spain; 4 Faculty of Social and Labor Sciences, Department of Psychology and Sociology, University of Zaragoza, Zaragoza, Spain; 5 Faculty of Medicine, Department of Medicine, Psychiatry and Dermatology, University of Zaragoza, Zaragoza, Spain; Universita degli Studi della Campania Luigi Vanvitelli, ITALY

## Abstract

Late-life cognitive decline ranges from the mildest cases of normal, age-related change to mild cognitive impairment to severe cases of dementia. Dementia is the largest global burden for the 21^st^ century welfare and healthcare systems. The aim of this study was to analyze the neuropsychological constructs (temporal orientation (TO), spatial orientation (SO), fixation memory (FM), attention (A), calculation (C), short-term memory (STM), language (L), and praxis (P)), semantic fluency, level of functionality, and mood that reveal the greatest deficit in the different stages ranging from normal cognition (NC) to cognitive impairment in older adults in a primary healthcare setting. The study included 337 participants (102 men, 235 women), having a mean age of 74 ± 6 years. According to their scores on the Spanish version of the Mini-Mental State Examination (MEC-35), subjects were divided into 4 groups: no deterioration (ND) (score 32–35), subtle cognitive impairment (SCI) (score 28–31), level deterioration (LD) (score 24–27) and moderate deterioration (MD) (score 20–23). The ND group revealed significant differences in TO, STM, C, A, L, P, and S-T as compared to the other groups. The MD group (in all the neuropsychological constructs) and the ND and SCI groups showed significant differences on the Yesavage geriatric depression scale (GDS-15). All except the FM neuropsychological construct were part of the MEC-35 prediction model and all of the regression coefficients were significant for these variables in the model. Furthermore, the highest average percentage of relative deterioration occurs between LD and MD and the greatest deterioration is observed in the STM for all groups, including A and TO for the LD and MD groups. Based on our findings, community programs have been implemented that use cognitive stimulation to prevent cognitive decline and to maintain the neuropsychological constructs.

## Introduction

Aging is a multifactorial process having modifiable and non-modifiable risk factors [1]. Modifiable risk factors have been defined by several authors who have classified them into sociodemographic, environmental [2], clinical, lifestyle [2,3], and cognitive [3] groups. Age is the most important socio-demographic risk factor for cognitive decline [4]. Old age is the greatest predictor of decreases in attention, memory, and global cognition [4]. Lifestyle-related factors have also been associated with cognitive impairment [5]. Clinical factors may include vascular risk factors that increase the risk of vascular dementia and Alzheimer’s disease (AD) and accelerate associated cognitive decline [6]. Cognitive decline in later life has numerous causes, and each may be associated with different risk or protective factors [7]. The improvement of modifiable risk factors and cognitive stimulation (CS), however, are considered effective means of ensuring healthy aging [1]. Late-life cognitive decline ranges from the mildest cases of normal, age-related change to mild cognitive impairment (MCI) to severe dementia [8].

Normal aging tends to be accompanied by complaints regarding the ability to acquire, consolidate and recall new information. Perception, processing speed, attention, and memory all tend to deteriorate in normal aging. Moreover, regarding memory, encoding is negatively affected, with impoverished memory representations being observed [9]. Cognitive problems in MCI include difficulties in memory, language, attention, orientation, calculation, visuospatial abilities, and executive functions, while the language is preserved [10]. Reisberg et al. (2008) [11] proposed that subjective cognitive impairment (SCI) may exist for up to 15 years before the deficits associated with MCI are reliably detected by practitioners. Progression rates from NC to MCI due to AD range from 4% to 10% annually [12].

MCI describes a stage of intermediate cognitive dysfunction where the risk of conversion to dementia is increased [13]. Memory failure is a predictor of future dementia in MCI [14]. The ability to execute complex instrumental activities of daily living (IADLs) may also be an important factor in differentiating between NC, MCI, and dementia [15]. However, the maintenance of basic activities of daily living is a critical factor for distinguishing between individuals with dementia and individuals with MCI [15]. In addition, an association exists between MCI and the possibility of suffering from concomitant depression or anxiety disorders [16]. It has been reported that 10–15%, 60.5% and 100% of all MCI patients will develop full dementia within 1 year, 5 years and 9.5 years, respectively, after their initial diagnosis with MCI [17].

Dementia is the greatest global burden on the 21^st^ century welfare and healthcare systems. The mean standard deviation (SD) for indirect and informal care costs per patient with AD living in the community in Spain over 6 months were estimated at €32,177.3 (€31,836.9) [18].

In this study, we have evaluated and compared the cognitive differences and semantic fluency in activities of daily living (ADLs) and the mood of older adults living in the community, classifying them into four groups based on cognitive level, according to the Spanish version of the Mini-Mental State Examination (MEC-35). Scores of over 27 points on the MEC-35 indicate an absence of cognitive impairment. However, scores of less than 27 points on the MEC-35 appear to indicate the presence of cognitive impairment [19]. The no deterioration (ND) group consists of older adults having scores between 32 and 35 points on the MEC-35, and the subtle cognitive impairment (SCI) group had scores between 28 and 31; the cut-off of 31 points on the MEC-35, corresponding to a score of 25 on the MMSE, is based on the classification of Friedman et al. 2012 [20]. The level deterioration (LD) group had scores between 24 and 27 on the MEC-35, in accordance with the classification by Calero et al. (2006) for individuals with MCI [19]. And the moderate deterioration (MD) group had scores ranging from 20 to 23, in accordance with Vinyoles Bargalló et al. 2002 [21] in the presence of cognitive impairment. The ND group indicated NC and the SCI group could indicate pre-symptomatic levels of cognitive impairment and decreased cognitive functioning [22]. The LD group could indicate MCI and the MD group could indicate mild dementia.

As far as we know, no studies have yet compared the cognitive construct at four levels. Few studies have examined the decline in discrete neuropsychological constructs as individuals progress from NC to MCI or cognitive impairment [23,24]. Other studies have been conducted at a single level on subjects receiving a diagnosis of MCI or dementia [24–27]. A work by Stites et al. [28] examined the relationship between the self-reporting of cognitive complaints and the quality of life in three groups (NC, MCI, and AD dementia), but did not explore neuropsychological constructs. Therefore, the aim of this study was to analyze the neuropsychological constructs (temporal orientation (TO), spatial orientation (SO), fixation memory (FM), attention (A), calculation (C), short-term memory (STM), language (L), and praxis (P)), semantic fluency, level of functionality, and mood that reveal the greatest deficit in the different stages ranging from normal cognition (NC) to cognitive impairment in older adults in a primary healthcare setting.

Our hypothesis states that certain neuropsychological constructs such as SO, L, and P are maintained even in participants demonstrating an established degree of impairment; however, STM, A, and TO, could show higher levels of impairment in older adults with cognitive impairment than those older adults without cognitive impairment, as compared to other neuropsychological constructs such as SO, L and P. Research has supported this hypothesis based on cognitive reserve (CR). CR can be achieved through an active cognitive lifestyle, which involves participating in cognitively stimulating activities that contribute to the delay or attenuation of symptoms related to brain damage and reduce the risk of dementia [29]. It would be interesting to predict which neuropsychological constructs are maintained and which worsen in the continuum without cognitive impairment as compared to that with impairment, so as to offer personalized therapeutic CS-based interventions adapted to the cognitive level of older adults.

## Materials and methods

This descriptive observational study was conducted in a Primary Care Centre in the city of Zaragoza (northeastern Spain). The sample consisted of 337 participants who were patients in primary healthcare consultations and received normal medical and nursing care.

Participants received information on the project from informative posters placed on the doors of all the medical consultation rooms and where their family doctors worked.

In order to detect the proportion of individuals having a certain level of cognitive impairment (as a four-category qualitative variable), the sample size was calculated for an expected proportion of 30%, with a 5% error and 95% confidence level. An algorithm implemented in WinEpi 2 was used for this calculation and an unknowing reference population has been assumed [30].

The inclusion criteria were: ≥ 65 years of age, receiving a score on the Spanish version of the Mini-Mental State Examination (MEC-35) ranging from 20 to 35 points, classified into 4 groups: between 32–35 points for the ND group; between 28–31 points for the SCI group; between 24–27 points for the LD group; and between 20–23 points for the MD group. The exclusion criteria were institutionalization, deafness, blindness neuropsychiatric disorders, motor difficulties, and having received CS over the past 12 months.

### Variables

Socio-demographic, clinical, lifestyle, contextual, and environmental variables were examined.

The socio-demographic variables studied were: age, gender, civil status, education level, physical occupational status, mental occupational status, and nucleus of family coexistence. Moreover, education level was divided into two subgroups (Primary/Higher). This is the most basic classification possible, given that this variable was not initially considered for the inference analysis of the results. The subdivision of physical occupational status and mental occupational status was made according to three levels: low, medium, and high for each, in accordance with the classification by Grotz et al. 2017 [31].

The clinical state variables examined were: high blood pressure (HBP), diabetes, hypercholesterolemia, obesity, heart disease, lung disease, peripheral vascular disease, visual disturbance, hearing impairment, cerebrovascular accident (CVA), alcoholism diagnosis, anxiety diagnosis, anxiety treatment, depression diagnosis, and depression treatment.

The lifestyle, contextual, and environmental variables studied were: physical activity, smoking, subjective perception of stress, interests, roles, values, ramp use, lift use, and showering.

For variable collection, over two weeks, trained occupational therapists administered an interview to all of the participants in which the different questions were answered; either with “yes” or “no” if they were questions with two-answer options or with the answer chosen from the distinct options presented in the case of questions with three or more response options.

Furthermore, the division of the subgroups was made in accordance with the level of physical activity (Sedentary lifestyle/Light/Moderate/Vigorous) for low, moderate and high activity levels, according to the International Physical Activity Questionnaire (IPAQ). Participants who did not perform any physical activity were included in the “Sedentary lifestyle” category [32]. Interests (Without interest/From 1 to 3 interests/More than 3 interests) roles (No role/One role/Two roles/Three roles/Four roles/Five roles/Six roles/None) and values (Health, happiness, peace, and tranquility/Family/Love and friendship/Human values/Culture, hope and religion/Independence) were based on a quantitative classification depending on the participants’ responses, in accordance with Gary Kielhofner (2011) [33]. These values relate to the development of abilities and skills connected to daily routines found in occupational performance [34].

### Neuropsychological assessment

The primary variable was the MEC-35, one of the most widely-used short cognitive tests for the study of cognitive capacities in Primary Care. It evaluates eight components: temporal and spatial orientation (10 points), fixation memory (3 points), attention (3 points), calculation (5 points), short-term memory (3 points), language, and praxis (11 points). Its sensitivity is 85–90% and its specificity is 69%. This questionnaire was used to assess the global cognition and cognitive functions of TO, SO, FM, STM, C, A, L, and P. Unlike the MMSE, the MEC-35 includes a three-digit series to repeat two similar items in reverse order. Subtraction is performed three by three from 30, instead of 7 by 7 from 100, as in the version by Folstein et al.1975 [35]. In this version, as the number of items increases, the maximum score reaches 35 points as compared to 30 in the original one [36]. For the cut-off point 24/27, the sensitivity and specificity of the MEC-35 have been described in 89.8% and 83.9%, respectively [19,21].

The secondary outcomes variables were Set-Test (S-T), Barthel Index (BI), Lawton and Brody scale (L-B), Goldberg anxiety sub-scale, and Yesavage geriatric depression scale, 15-point version (GDS-15).

Semantic fluency was measured with the S-T in four categories: colors, animals, fruits, and cities. Scores range from 0 to 40, with 0 being the minimum and 40 being the maximum score. This test has been proposed as a diagnostic aid in elderly patients with dementia, having a cut-off of 27 points for the elderly, with a lower score indicating dementia. This test has a documented sensitivity of 79% and a specificity of 82% [37].

The independence in ten basic activities of daily living (BADLs) was evaluated with the BI. The maximum score is 100 points and scores ≥ 60 indicate mild dependence. The sensitivity of this test ranges from 76% (in the item “ambulation + stairs”) to 99.8% (in the item “feeding”) and its specificity ranges from 46% (in the item “defecation”) and 97% (in the item “ambulation + stairs”) in scores ≥ 90 points for fragility screening [38].

The autonomy in eight instrumental activities of daily living (IADLs) necessary to live independently was assessed with the L-B. Scores range from 0 (dependent) to 8 (independent). The scale’s sensitivity is 57% and its specificity is 82% when an informant observes dependence in three activities [39].

Anxiety was measured using the Goldberg anxiety sub-scale, which is a sub-scale of the Goldberg questionnaire, with nine dichotomous response items (yes/no responses). An independent score is awarded for each scale, with one point for an affirmative answer. The cut-off value is ≥ 4 for the anxiety sub-scale, indicating “probable anxiety”. This scale has a specificity of 91% and a sensitivity of 86% [40].

The depression level was evaluated with the GDS-15 and is considered suitable for seniors in the community. Scores range from 0 to 15, with a total score > 5 interpreted as “probable depression”. In older adults, with a cut-off of 5 points, sensitivity is 71.8% and specificity is 78.2% [41].

The evaluation process was performed by occupational therapists after receiving the corresponding training to ensure the homogeneous application of evaluation instruments.

### Statistical analysis

The statistical analysis was performed with the IBM SPSS Statistics Package, v.22. The descriptive statistics are shown according to the nature of each variable. For the quantitative variables, the mean ($\bar{x}$), SD, and 95% confidence interval level were used for the population mean. Due to the non-symmetry of some of these variables, we also included the median (Me), the first (Q1) and third (Q3) quartile and the extreme points (Table 2). For qualitative variables, the number of participants in each category (n) and the proportion of patients over the total (%) were considered. The Kolmogorov-Smirnov test was used to verify the normality of the quantitative variables. Most of them are non-normal distributions.

The Pearson Chi-square test was used to determine associations between qualitative variables. Differences between groups in the cognitive measurements were evaluated using the non-parametric Mann-Whitney U test. In addition, Spearman correlation coefficients were calculated between the cognitive measurements and the ANOVA analysis was used for predictive multiple linear regression models.

### Ethical considerations

This study was approved by the Research Ethics Committee of the Autonomous Community of Aragón, protocol number (CEICA PI11/90 and PI11/00091). All personal data protection regulations were respected. Participants were informed of the study objectives and they signed a written informed consent. The deontological norms recognized by the Declaration of Helsinki (52^nd^ WMA General Assembly, Edinburgh, Scotland, October 2000) [42] and good clinical practice norms were followed, and current legislation was complied with.

## Results

This study included 337 older adults with MEC-35 scores between 20 and 35 points; 69.7% (235) were women and 30.3% (102) were men. Their mean age was 74, with an SD of 6.

No statistically significant differences were observed in socio-demographic characteristics, clinical characteristics, participants’ lifestyle, contextual and environmental variables (Table 1). The profile was a married (67.4%) woman (69.7%) living with her partner (56.7%), having a primary education level (79.8%), a medium physical occupation (60.8%) and a low mental occupation (43%). A major difference was observed between the percentage of men and women. On the one hand, in the region where the study took place, and in Spain in general, the percentage of women over the age of 75 is 60% higher than that of men (68% for those over the age of 80). On the other hand, it is a cultural fact that Spanish women tend to be more participative and more consistent in their participation when collaborating in this type of studies. Men tend to refuse to participate and are much less consistent. Therefore, it is difficult to obtain a larger male sample.

**Table 1:** The participants’ socio-demographic and clinical characteristics and participants’ lifestyle, contextual and environmental variables.

		Total (n = 337)						
Age (years) Mean(SD)		74 (6)						
Participants’ socio-demographic characteristics		n (%)	Participants’ clinical characteristics		n (%)	Participants’ lifestyle, contextual and environmental variables		n (%)
**Gender**	Men	102 (30.3)	**High blood pressure**	No	163 (48.4)	**Physical activity**	Sedentary lifestyle	32 (9.5)
	Women	235 (69.7)		Yes	174 (51.6)		Light	34 (10.1)
**Civil Status**	Single	17 (5)	**Diabetes**	No	284 (84.3)		Moderate	240 (71.2)
	Widowed	7 (2.1)		Yes	53 (15.7)		Vigorous	31 (9.2)
	Married	227 (67.4)	**Hypercholesterolemia**	No	212 (62.9)	**Smoking**	No	328 (97.3)
	Separated	86 (26.5)		Yes	125 (37.1)		Yes	9 (2.7)
**Education level**	Primary	269 (79.8)	**Obesity**	No	286 (84.9)	**Subjective perception of stress**	No	282 (83.7)
	Higher	68 (20.2)		Yes	51 (15.1)		Yes	55 (16.3)
**Physical occupational status**	Low	63 (18.7)	**Heart disease**	No	267 (79.2)	**Interests**	No interests	39 (11.6)
	Medium	145 (43)		Yes	70 (20.8)		From 1 to 3 interests	212 (62.9)
	High	129 (38.3)	**Lung disease**	No	298 (88.4)		More than 3 interests	86 (25.5)
**Mental occupational status**	Low	205 (60.8)		Yes	39 (11.6)	**Roles**	No role	4 (1.2)
	Medium	112 (33.2)	**Peripheral vascular disease**	No	242 (71.8)		One role	148 (43.9)
	High	20 (5.9)		Yes	95 (28.2)		Two roles	135 (40.1)
**Nucleus of family coexistence**	Living alone	65 (19.3)	**Visual disturbance**	No	63 (18.7)		Three roles	36 (10.7)
	Living with partner	191 (56.7)		Yes	274 (81.3)		Four roles	10 (3)
	Living with children	29 (8.6)	**Hearing impairment**	No	212 (62.9)		Five roles	2 (0.6)
	Living with partner and children	33 (9.8)		Yes	125 (37.1)		Six roles	2 (0.6)
	Living with children and grandchildren	3 (0.9)	**Cerebrovascular accident**	No	315 (93.5)	**Values**	None	9 (2.7)
	Living with partner, children, and grandchildren	3 (0.9)		Yes	22 (6.5)		Health, happiness, peace, and tranquility	157 (46.6)
	Living with grandchildren	13 (3.9)	**Alcoholism diagnosis**	Yes	1 (0.3)		Family	113 (33.5)
				No	261 (77.4)		Love and friendship	29 (8.6)
			**Anxiety diagnosis**	Yes	76 (22.6)		Human values	24 (7.1)
				No	273 (81)		Culture, hope, and religion	3 (0.9)
			**Anxiety treatment**	Yes	64 (19)		Independence	2 (0.6)
				No	271 (80.4)	**Ramp use**	No	2 (0.6)
			**Depression diagnosis**	Yes	66 (19.6)		Yes	181 (53.7)
				No	283 (84)	**Lift use**	No	156 (46.3)
			**Depression treatment**	Yes	54 (16)		Yes	43 (12.8)
				No	163 (48.4)	**Showering**	No	294 (87.2)
							Yes	130 (38.6)

Table 2 presents the descriptive study of the quantitative variables of participants by groups. In the MEC-35, the mean for the ND group was 33.21 points, the mean for the SCI group was 29.45 points, for the LD group was 25.79 points, and for the MD group, 21.89 points. As for neuropsychological constructs, a trend was observed between groups of greater impairment in STM, A, and C. Large differences were not found between groups in ADLs, with all groups having normal values for mood.

**Table 2:** By levels, descriptive of the quantitative variables.

	**Variables**	***Mean***	***Std***	***IC***	***Q1***	***Me***	***Q3***	***Min***	***Max***
	**MEC-35**	33.21	1.01	33–33.41	32	33	34	32	35
	**Cognitive aspects**								
**NO DETERIORATION GROUP**	*Temporal orientation*	4.85	0.4	4.77–4.93	5	5	5	3	5
	*Spatial orientation*	4.88	0.35	4.8–4.95	5	5	5	3	5
	*Short-Term memory*	2.35	0.78	2.19–2.5	2	2	3	0	3
	*Fixation memory*	3	-	-	-	-	-	-	-
	*Calculation*	4.89	0.34	4.82–4.96	5	5	5	3	5
	*Attention*	2.66	0.79	2.51–2.82	3	3	3	0	3
	*Language*	5.77	0.44	5.68–5.86	6	6	6	4	6
	*Praxis*	4.8	0.42	4.72–4.89	5	5	5	3	5
	**Set-Test**	39.05	1.63	38.73–39.37	39	40	40	32	40
	**Barthel**	97.67	5.4	96.6–98.74	97.5	100	100	65	100
	**Lawton**	7.3	1.15	7–7.52	7	8	8	3	8
	**Goldberg**	2.09	1.74	1.59–2.59	0.5	3	5	0	6
	**GDS-15**	1.34	1.6	0.87–1.8	1	2	3.5	0	7
	**MEC-35**	29.45	1	29.25–29.65	29	29.5	30	28	31
	**Cognitive aspects**								
**SUBTLE COGNITIVE IMPAIRMENT GROUP**	*Temporal orientation*	4.56	0.73	4.42–4.7	4	5	5	1	5
	*Spatial orientation*	4.8	4.7	4.71–4.89	5	5	5	3	5
	*Short-Term memory*	1.6	0.97	1.4–1.8	1	2	2	0	3
	*Fixation memory*	3	-	-	-	-	-	-	-
	*Calculation*	4.54	0.7	4.4–4.68	4	5	5	2	5
	*Attention*	1.34	1.14	1.11–1.57	1	1	3	0	3
	*Language*	5.25	0.78	5–5.41	5	5	6	3	6
	*Praxis*	4.36	0.7	4.22–4.5	4	4	5	2	6
	**Set-Test**	37.82	3.13	37.2–38.4	37	39	40	26	40
	**Barthel**	97.23	5.28	96.18–98.27	95	100	100	75	100
	**Lawton**	7.32	1.21	7–7.56	7	8	8	2	8
	**Goldberg**	3	2.49	2.17–3.86	0	2	5	0	7.5
	**GDS-15**	2.4	2.4	1.57–3.24	1	2	4	0	12
	**Variables**	***Mean***	***Std***	***IC***	***Q1***	***Me***	***Q3***	***Min***	***Max***
	**MEC-35**	25.79	1	25.59–25.82	25	26	27	24	27
	**Cognitive aspects**								
**LEVEL DETERIORATION GROUP**	*Temporal orientation*	3.88	1.1	3.67–4.1	3.25	4	5	0	5
	*Spatial orientation*	4.35	0.7	4.21–4.49	4	4	5	2	5
	*Short-Term memory*	0.9	0.9	0.7–1.08	0	1	2	0	3
	*Fixation memory*	2.99	0.1	2.97–3.01	3	3	3	2	3
	*Calculation*	3.72	1.3	3.47–3.97	3	4	5	0	5
	*Attention*	1.08	1	0.88–1.29	0	1	1	0	3
	*Language*	4.69	0.9	4.5–4.86	4	5	5	2	6
	*Praxis*	4.14	0.77	3.99–4.29	4	4	5	2	5
	**Set-Test**	35.65	4.7	34.75–36.5	34	37	39	21	40
	**Barthel**	95.97	7	94.63–97.32	95	100	100	65	100
	**Lawton**	6.87	1.7	6.54–7.2	6	8	8	0	8
	**Goldberg**	3	2.58	2.2–3.8	1	3	5	0	9
	**GDS-15**	3.38	3.47	2.3–4.45	1	2.25	4.87	0	12
	**MEC-35**	21.89	1.1	21.47–22.32	21	22	23	20	23
	**Cognitive aspects**								
**MODERATE DETERIORATION GROUP**	*Temporal orientation*	2.79	1.3	2.29–3.28	2	2.5	4	0	5
	*Spatial orientation*	4.29	0.9	3.95–4.63	4	4.5	5	2	5
	*Short-Term memory*	0.36	0.56	0.14–0.57	0	0	1	0	2
	*Fixation memory*	3	-	-	-	-	-	-	-
	*Calculation*	2.5	1.35	1.98–3	1.25	3	3	0	5
	*Attention*	0.89	1	0.51–1.28	0	1	1	0	3
	*Language*	4.32	1.16	3.87–4.77	3	4	5	2	6
	*Praxis*	3.75	0.8	3.44–4.1	3	4	4	2	5
	**Set-Test**	31.29	5.28	29.2–33.3	27.5	32	35	21	40
	**Barthel**	92.32	7.4	89.46–95.19	85	90	100	80	100
	**Lawton**	6.36	1.8	5.65–7	4.25	7	8	3	8
	**Goldberg**	3	2.5	-	1	2,25	5.75	0	8
	**GDS-15**	4.34	3.9	-	0.625	3.5	7.5	0	12

IC: 95% Confidence Interval level for the population mean; Me: Median; Q1, Q3: First and third quartile; Goldberg: Goldberg anxiety sub-scale; GDS-15: Yesavage geriatric depression scale, 15-point version; MEC-35: Spanish version of the Mini-Mental State Examination.

Table 3A presents the comparison by levels for all of the quantitative variables. The following aspects are of interest: As for the neuropsychological constructs, the ND group revealed significant differences as compared to the rest of the groups in the MEC-35, TO, STM, C, A, L, P, and S-T. No significant differences were observed in SO for the SCI group, as was the case for the variables BI and L-B. With respect to the LD group, only L-B prevails without differences. As anticipated, regarding the MD group, significant differences were found for all neuropsychological constructs. For the SCI group, however, no differences were observed with the level deterioration and moderate deterioration groups in A. The same pattern can be observed for BI and L-B, where L-B was once again the last to reveal the differences. Finally, between the LD and moderate deterioration groups, more cognitive variables without significant differences were found, such as SO, A, L, and L-B. As for the emotional aspects, significant differences in the GDS-15 were only found between the ND and SCI groups.

**Table 3a1:** By levels, the p-value of the mean differences test between the quantitative variables by gender.

	MEC-35	TO	SO	STM	C	A	L	P	S-T	Barthel	Lawton	Goldberg	GDS-15
ND	0.644	0.878	0.846	0.091	0.248	0.589	0.912	0.967	0.081	0.574	**	0.011*	0.002*
SCI	0.043*	0.076	0.107	0.012*	**	0.562	0.136	0.382	0.275	0.400	**	0.018*	0.001*
LD	0.144	0.125	0.287	0.031*	0.022*	0.071	0.720	0.719	0.335	0.027*	**	0.217	0.373
MD	0.022*	0.550	0.519	0.002*	0.739	0.122	0.719	0.779	0.175	0,326	0.038*	0.029*	**

MEC-35: Spanish version of the Mini-Mental State Examination; ND: No deterioration group; SCI: Subtle cognitive impairment group; LD: Level deterioration group; MD: Moderate deterioration group; TO: Temporal Orientation; SO: Spatial Orientation; STM: Short Term Memory; C: Calculation; A: Attention; L: Language; P: Praxis; S-T: Set-Test of semantic fluency; Barthel: Barthel index; Lawton: Lawton and Brody scale; Goldberg: Goldberg anxiety sub-scale; GDS-15: Yesavage geriatric depression scale, 15-point version. Differences are contrasted with the Mann-Whitney Test at every two different levels.

** and * mean p-value <0.001, <0.05 respectively.

**Table 3a2:** By levels and gender, the p-value of the mean differences test between the quantitative variables.

			MEC-35	TO	SO	STM	C	A	L	P	S-T	Barthel	Lawton	Goldberg	GDS-15
ND	SCI	M	**	**	0.05	**	**	**	0.027*	**	0.008*	0.428	0.236	0.115	0.934
		W	**	0.014*	0.055	**	**	**	**	**	0.05*	0.211	0.058	0.253	0.967
	LD	M	**	**	**	**	**	**	**	**	**	0.736	0.071	0.564	0.051
		W	**	**	**	**	**	**	**	**	**	0.017*	**	0.508	0.802
	MD	M	**	**	**	**	**	**	**	**	**	0.322	0.127	0.431	0.520
		W	**	**	**	**	**	**	**	**	**	**	**	0.810	0.033*
SCI	LD	M	**	0.028*	0.325	0.029*	0.026*	0.984	**	0.652	0.051	0.693	0.516	0.041*	0.093
		W	**	**	**	**	**	0.039*	**	0.031*	**	0.198	0.049*	0.554	0.857
	MD	M	**	0.029*	0.561	0.011*	**	0.065	0.015*	0.230	0.003*	0.251	0.158	0.743	0.494
		W	**	**	**	**	**	0.302	0.003*	**	**	0.002*	**	0.312	0.049*
LD	MD	M	**	0.137	0.937	0.064	0.004*	0.034*	0.295	0.291	0.033*	0.161	0.317	0.258	0.082
		W	**	**	0.982	0.020*	0.002*	0.736	0.222	0.033*	**	0.027*	0.022*	0.566	0.045*

MEC-35: Spanish version of the Mini-Mental State Examination; ND: No deterioration group; SCI: Subtle cognitive impairment group; LD: Level deterioration group; MD: Moderate deterioration group; TO: Temporal Orientation; SO: Spatial Orientation; STM: Short Term Memory; C: Calculation; A: Attention, L: Language; P: Praxis; S-T: Set-Test of semantic fluency; Barthel: Barthel index; Lawton: Lawton and Brody scale; Goldberg: Goldberg anxiety sub-scale; GDS-15: Yesavage geriatric depression scale, 15-point version. Differences are contrasted with the Mann-Whitney Test at every two different levels.

** and * mean p-value <0.001, <0.05 respectively.

**Table 3b:** Percentages of the relative deterioration between consecutive levels.

	TO	SO	STM	C	A	L	P	Average
No deterioration—Subtle cognitive impairment	6.6	1.8	31.9	7.2	49.6	9	9.2	14.4
Subtle cognitive impairment—Level deterioration	14.9	9.4	43.7	18	19.4	10.7	5	15.2
Level deterioration—Moderate deterioration	28	1.4	60	32.8	17.6	7.9	9.4	19.6

TO: Temporal Orientation; SO: Spatial Orientation; STM: Short-Term Memory; C: Calculation; A: Attention; L: Language; P: Praxis.

From these data, we calculated the relative deterioration for each group with respect to the next for all of the cognitive constructs. Table 3B shows the percentage as well as the average of relative deterioration. The highest average percentage of relative deterioration occurs between the LD and MD groups. The greatest deterioration is observed in STM for all of the groups, and in A and TO for the LD and MD groups.

For the quantitative variables, the differences between these groups have been analyzed. First, no differences in the age variable have been found with regard to gender (men: 74.5, women: 74; p-value: 0.260), and therefore the possible age interaction has been ruled out. Next, quantitative differences based on gender were examined: Table 3A_1_ presents the p-value of the mean differences test between the quantitative variables by gender, according to level. No differences were found for any variable in the ND group. Differences were found in STM in the other three groups, in C, in the SCI and LD groups, and in the MEC-35 in the SCI and ND groups. For purposes of precision, a new Table 3A_2_ was created presenting the differences between the quantitative variables while stratifying the study by gender.

Table 4 analyzes the correlation coefficients between the different outcome variables. The following positive correlations having statistically significant differences are observed: the MEC-35 with all neuropsychological constructs (TO, SO, STM, C, A, L, and P); S-T with BI and L-B; TO with SO, STM, C, L, P, S-T, BI, and L-B; SO with STM, P, S-T, and L-B; STM with C, L, P, A, S-T, BI, and L-B; C with A, L, P, S-T, and BI; A with L and P; L with P, S-T, and BI; BI with L-B; and L-B with Goldberg. And the following negative correlations were found, also with significant differences: C with GDS-15; BI with Goldberg and GDS-15; and Goldberg with GDS-15. In general, we can affirm that all of the cognitive variables are positively correlated. This conclusion, however, cannot be made for the cognitive variables and their relationship with the daily living and mood variables.

**Table 4:** Correlation coefficients between quantitative variables. The significant correlations are marked.

	MEC-35	TO	SO	STM	FM	C	A	L	P	S-T	Barthel	Lawton	Goldberg	CDS-15
MEC-35	-	0.593**	0.388**	0.633**	0.041	0.590**	0.528**	0.569**	0.478**	0.493**	0.198**	0.169**	-0.12	-0.1
TO	-	-	0.235**	0.353**	0.015	0.275**	0.079	0.240**	0.168**	0.408**	0.181**	0.219**	0.107	-0.046
SO	-	-	-	0.175**	0.056	0.105	0.063	0.079	0.175**	0.321**	0.088	0.114*	-0.063	-0.82
STM	-	-	-	-	0.074	0.198**	0.128*	0.260**	0.192**	0.379**	0.109*	0.218**	0.033	-0.027
FM	-	-	-	-	-	0.01	0.028	-0.05	0.027	-0.027	-0.030	0.003	0.042	-0.098
C	-	-	-	-	-	-	0.252**	0.281**	0.165**	0.257**	0.173**	-0.030	-0.048	-0.142**
A	-	-	-	-	-	-	-	0.199**	0.215**	0.080	0.061	-0.070	-0.065	-0.05
L	-	-	-	-	-	-	-	-	0.162**	0.235**	0.214**	0.089	0.016	-0.019
P	-	-	-	-	-	-	-	-	-	0.254**	-0.057	0.071	-0.015	-0.001
S-T	-	-	-	-	-	-	-	-	-	-	0.118*	0.205**	0.040	-0.056
Barthel	-	-	-	-	-	-	-	-	-	-	-	0.290**	-0.202**	-0.311**
Lawton	-	-	-	-	-	-	-	-	-	-	-	-	0.160**	-0.011
Goldberg	-	-	-	-	-	-	-	-	-	-	-	-	-	-0.523**
CDS-15	-	-	-	-	-	-	-	-	-	-	-	-	-	-

MEC-35: Spanish version of the Mini-Mental State Examination; TO: Temporal Orientation; SO: Spatial Orientation; STM: Short Term Memory; FM: Fixation memory; C: Calculation; A: Attention; L: Language; P: Praxis; S-T: Set-Test of semantic fluency; Barthel: Barthel index; Lawton: Lawton and Brody scale; Goldberg: Goldberg anxiety sub-scale; GDS-15: Yesavage geriatric depression scale, 15-point version. Spearman correlation coefficient is given for every two quantitative variables.

** and * mean p-value <0.001, <0.05 respectively.

Table 5 shows the regression models for the prediction of the MEC-35, S-T, Barthel, and Lawton variables, in terms of TO, SO, STM, C, A, L, P, Goldberg, and GDS-15 variables. The regression coefficients and their significance are shown in the table, and it is evident that all of the neuropsychological constructs participate in the MEC-35 prediction, while TO, SO, STM, C, and P take part in the S-T prediction.

**Table 5:** Regression models.

	*R* ^2^	Constant	TO	SO	STM	C	A	L	P	Goldberg	CDS-15
MEC-35	0.095**	3.209**	1.013**	0.958**	0.982**	0.959**	1.006**	1.005**	1.023**	0.003	-0.001
S-T	0.359**	17.927**	1.310**	1.045*	0.561*	0.426*	-0.035	0.413	0.862**	0.052	-0.019
Barthel	0.207**	90.854**	0.615	0.605	-0.125	0.365	0.057	1.1142*	-1.027*	-0.153	-0.716**
Lawton	0.175**	3.839**	0.353**	0.235	0.153*	-0.042	-0.115	0.052	0.061	0.106*	-0.020

MEC-35: Spanish version of the Mini-Mental State Examination; TO: Temporal Orientation; SO: Spatial Orientation; STM: Short Term Memory; C: Calculation; A: Attention; L: Language; P: Praxis; S-T: Set-Test of semantic fluency; Barthel: Barthel index; Lawton: Lawton and Brody scale; Goldberg: Goldberg anxiety sub-scale; GDS-15: Yesavage geriatric depression scale, 15-point version.

** and * mean p-value <0.001, <0.05 respectively.

In Barthel’s prediction, the variables L, P, and GDS-15 are significant, and we can highlight the last negative coefficients. Finally, the predictive model for L-B is significant through the linear combination of TO, STM, and Goldberg variables.

## Discussion

This study explored the neuropsychological constructs, functionality, and mood according to the cognitive level in four groups of older adults attending a primary healthcare center in Spain. We have shown that older adults in the continuum without cognitive impairment had poorer performance on the neurological constructs of STM, A, and TO, as compared to the moderate cognitive impairment group. Therefore, it would be interesting to personalize CS-based therapeutic interventions, adapting them to the participants’ specific cognitive level.

Since the prediction model includes all neuropsychological constructs except for FM, and all of the regression coefficients are significant, it is evident that the ability to identify older adults at risk for developing AD increases when all of these constructs are included in the assessed tasks [43–45].

Other studies are in line with our findings on memory. In a study by Mistridis et al. 2015 [46], memory declined in the initially healthy participants with subsequent MCI relative to the demographically-matched healthy group. MCI subjects [24,47–50], subjects in the preclinical period in AD [25,51], and subjects with dementia due to AD [52,53] present a decreasing tendency in memory. In short, memory deficits are good predictors of conversion from: 1) NC to MCI [54]; 2) MCI to AD [48,55]. The ability to maintain attention is essential [56]. Without it, other cognitive functions would be compromised [57]. Attention differs from the other cognitive functions because it requires significant subjective effort [58]. Also, people are less able to maintain their attention as they age, which could explain the attention gaps suffered by those with SCI [59] as well as the negative findings found in the change of level from NC to SCI with respect to attention in our study. Similar results regarding attention were found in other studies, with regard to the other groups. In healthy aging, there is an increased presence of compensatory interactions between attention networks that may be no longer effective and the emergence of clinical symptoms in MCI. These may serve as cognitive markers in individuals at an increased risk of developing AD [60]. MCI subjects [37,50] and patients with AD [53,61] reveal a decreasing tendency in attention. In other studies, cognitive changes occurring with NC indicate that the attention processes have been negatively affected [62,63]. As in our study, the 2016 Commodari study [64] observed gender differences in the participants’ cognitive function “attention” based on the level of cognitive functioning.

Other research found similar results in TO. TO is a component used to diagnose cognitive impairment. It is among the first neuropsychological constructs to be impaired in AD [65]. TO presents a greater difference between subjects with MCI or dementia and those without impairment [66]. Other studies, however, have observed that deterioration of SO in MCI is associated with a higher risk of progression to AD [67] and spatial disorientation is common in AD [68].

Moreover, individuals with MCI or subjective memory complaints who progress to dementia had poorer performance as compared to individuals that did not progress to dementia, according to a range of neuropsychological constructs such as memory and attention [69]. In addition, Batum et al. [48] revealed that the diagnosis of MCI should be established when attention, orientation, and long-term memory are affected.

Although normal values for the S-T are observed in the 4 groups, the tendency decreases with cognitive deterioration. In line with our results, other authors have noted that changes in semantic fluency may precede general cognitive decline and could help to predict AD [70]. The decline in semantic fluency during aging may originate from both semantic memory degradation and executive function deficits. In a recent meta-analysis that compared MCI participants with NC participants, the results suggest that the semantic network is preserved in MCI, however, existing associations are less efficiently exploited during long-term memory search, possibly due to deficits in the executive function [71]. In a recent study comparing participants with MCI and AD, it was observed that a significant interaction existed between the groups and the verbal fluency condition (phonemic and semantic). However, participants with AD produced significantly fewer words in both conditions whereas participants with MCI revealed a pattern similar to control subjects in the phonemic condition, but generated significantly fewer words in the semantic condition [72].

We observed very few differences for the ADLs in relation to the four groups. Other studies have found that impairment in ADLs is already present in MCI [73]. However, our findings indicate that these impairments may manifest even prior to the onset of clinical decline in NC [22,74]. In addition, the IADLs restrictions have an additional prognostic for subsequent dementia [74,75]. For mood, the four-level values in this study are within the limits of normality. Moreover, they appear to go in ascending order with cognitive deterioration. In other studies, depression and anxiety have been found to be typical of MCI [76] particularly, with depression being a predictor of the conversion of MCI to AD [76,77].

One strength of this study is that it considers from cognitive impairment to moderate cognitive impairment in order to make group comparisons and establish potential differences. Moreover, the total number of subjects was adequate and the participants recruited from Primary Healthcare allow us to extrapolate our results to the general population.

The study has several limitations. First, it used a cross design. However, when performing the four-group comparison, greater power was obtained for the results. Second, the Spanish version of MMSE has a known ceiling effect, with most of the NC participants obtaining the highest or closest possible score. Within the context of detecting turning points, older adults may begin some accelerated decline years before it is detected by the questionnaire. Thus, the turning points reported in published studies represent the endpoints of the ceiling effect rather than the true onset of the accelerated cognitive decline. Third, in this study, the anxiety and depression scales were selected given that they are short tests that evaluate seniors living in the community. The aim was to compare the differences established for the four groups of participants and to determine which groups had non-normal values. The Goldberg Anxiety sub-scale is a widely used instrument in the healthcare practice and in clinical research [78], however, it is often used as a screening test. Fourth, we have not found any studies that analyze the differences in the four groups of participants presented. Therefore, we had to make comparisons with articles that analyze a single group or that compare two groups. Fifth, the number of subjects included in the MD group was small as compared to the other groups. Therefore, additional research is needed in many subjects with a similar sample of participants per group (including participants with NC, SMC, MCI, and dementia) to examine the differences between the neuropsychological constructs, functionality, and mood based on the cognitive level.

## Conclusions

The results demonstrated the differences existing between the neuropsychological constructs, functionality, and mood based on the cognitive level in four groups of older individuals, in order to design personalized and adapted therapeutic interventions. As a result of our findings, we have implemented some community programs based on CS to prevent cognitive decline and to maintain the neuropsychological constructs. It would be of great interest to carry out a personalized intervention, adapting the stimulating activities to the life history, personal preferences, limitations, and potentialities of the patient [79]. And we must not forget that cognitive aspects such as STM, A, and TO suffer a greater deterioration in all participant groups, therefore, they should be reinforced in the interventions by including techniques of orientation to reality and external aid. CS refers to the set of techniques and strategies that attempt to optimize the performance of cognitive functions by compensation activities and strategies and CR to reinforce cerebral neuroplasticity [80]. Cognitive stimulating activities help to increase the CR, which has been shown to be a protective factor [81]. CR provides an explanation for the uneven predisposition to distinct age-related brain changes between older adults, while some subjects withstand these changes by maintaining their neuropsychological construct [82]. It has been postulated that individuals with greater reserve levels will cope with brain damage more successfully than those with low reserve levels [83] and therefore, a hypothesis would state that an increased cognitive reserve level may lead to a decline in the deterioration process [84]. In the meta-analysis by Colangeli et al. 2016 [85], it was commented that neural networks in CN patients remain intact; however, in patients with AD and MCI, these networks are no longer functional. Therefore, the brain activates other networks, through a compensation mechanism, to reorganize brain resources to cope with a cognitive task that otherwise, would be extremely difficult.
